# Perioperative Nursing Management of Unintended Retained Foreign Object–Related Events in Neurosurgery: Insights From a 16‐Year Study in China

**DOI:** 10.1155/jonm/5532006

**Published:** 2026-08-12

**Authors:** Chao Du, Shuo Zhang, Shenghua Lu, Dan Liu, Jing Li, Wei Wang, Hongling Jiao, Xiaonan Liu

**Affiliations:** ^1^ Department of Neurosurgery, Beijing Tiantan Hospital, Capital Medical University, Beijing, China, ccmu.edu.cn; ^2^ Beijing Neurosurgical Institute, Capital Medical University, Beijing, China, ccmu.edu.cn; ^3^ Department of Operating Room, Beijing Tiantan Hospital, Capital Medical University, Beijing 100070, China, ccmu.edu.cn

**Keywords:** adverse events, neurosurgery, nurse management, perioperative team, retained foreign object, surgical safety

## Abstract

**Background:**

This retrospective study aimed to investigate the frequency and characteristics of unintended retained foreign object (URFO)–related events in a single‐center neurosurgical operating room, focusing on surgical and intraoperative team–related variables to provide insights into improving operative safety and nursing management.

**Methods:**

We analyzed 543 URFO‐related events from 444,252 neurosurgical procedures performed between 2008 and 2024, based on data from the institutional adverse event reporting system. Events were categorized by type, and clinical, procedural, and perioperative team characteristics were analyzed using multinomial logistic regression to identify patterns associated with different categories of URFO‐related events.

**Results:**

URFO‐related events were classified as cotton material (*n* = 132, 24%), needle (*n* = 156, 29%), device fragments (*n* = 151, 28%), and device loss (*n* = 104, 19%). Cotton pads (Type S) and dura suture needles (Type 4‐0) were most frequently involved in their respective categories. Pattern analysis showed that URFO‐related events were more commonly observed in brain tumor surgeries, procedures lasting ≥ 6 h, and in operations involving relatively less experienced surgical and nursing staff.

**Conclusion:**

URFO‐related events were rare but represent important perioperative safety incidents reflecting potential near‐miss failures in surgical item management. These events demonstrated distinct procedural and team‐related patterns, particularly in complex and prolonged neurosurgical procedures. The findings highlight key risk‐prone situations within the operative workflow and support the need for strengthened perioperative management and standardized preventive processes to enhance surgical safety and quality of care.

**Implications for Nursing Management:**

Establishing standardized adverse event monitoring systems and fostering structured safety awareness and procedural compliance may strengthen operating room safety management, enhance perioperative risk control, and improve patient outcomes.

## 1. Introduction

Unintended retained foreign object (URFO)–related events, including retained or potentially retained cotton hemostatic materials, needles, and surgical instruments, are serious adverse events in neurosurgery. Previous studies have reported a frequency of approximately 3.56 per 10,000 surgeries [1, 2] While many foreign objects are identified before surgical closure and are therefore classified as near‐miss incidents [3], these events remain clinically significant. Their detection may prolong operative time, increase intraoperative complexity, and potentially elevate patient risk. In some cases, foreign objects may remain undetected, underscoring the responsibility of the surgical team to ensure that no items are retained within the patient.

**FIGURE 1:**
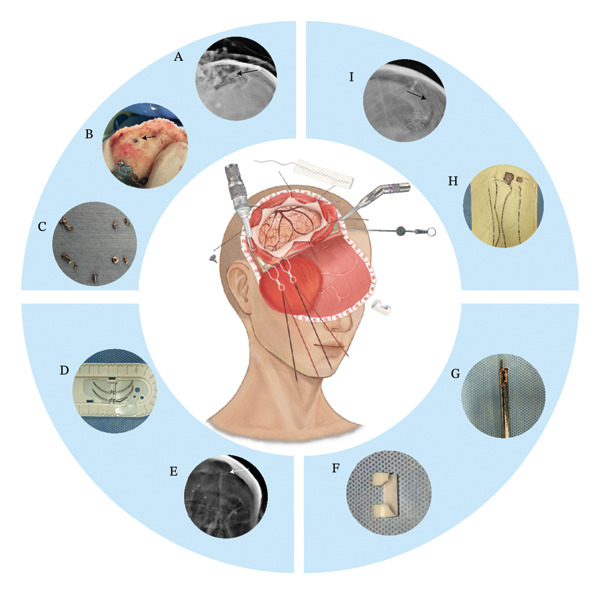
Intraoperative imaging and representative examples of URFO‐related events. (A–C) Titanium nails (lost items indicated by black arrows). (D, E) Needles (lost items indicated by white arrows). (F, G) Device fragments. (H, I) Cotton material (radiopaque markers indicated by black arrows).

Due to the intricate nature of neurosurgery, the likelihood of losing small items is higher compared to other surgical specialties. This poses a potential risk for neurological complications and, in severe cases [4], could even result in patient death [2]. Currently, there are no measures that can entirely prevent URFO‐related events. In addition to enhancing the management of operating room nurses and improving adverse event reporting, intraoperative imaging is the primary available solution (Figure 1A,E,I). However, intraoperative imaging has its limitations: It is effective primarily for detecting metallic objects, while it offers limited utility for nonradiologically sensitive materials [5, 6]. Furthermore, the sensitivity of intraoperative X‐ray imaging in detecting retained surgical items is only 67% [7].

Despite decades of advances in microneurosurgery, research focusing on URFO‐related events remains limited. Our study provides a comprehensive analysis of the perioperative characteristics and distribution patterns associated with URFO‐related events in neurosurgery, offering valuable evidence to support patient safety initiatives, optimize perioperative management practices, and improve healthcare quality. These findings may contribute to the refinement of hospital nursing management strategies and enhance the current understanding of the clinical features and operational patterns of URFO‐related events.

## 2. Methods

### 2.1. Data Collection and Statistics

In this retrospective descriptive study, we collected records of URFO‐related events from the operating room of our hospital between January 1, 2008, and December 31, 2024. A total of 543 URFO‐related events were identified during neurosurgical procedures, out of a total of 444,252 neurosurgical operations performed during this period.

During the data collection process, URFO‐related events were systematically classified, and seven relevant variables were grouped into predefined categories for analysis, including etiology, operation time, neurosurgeon seniority, night shift status, circulating nurse seniority, scrub nurse seniority, and multiple operations. Detailed definitions of all classifications are provided in the supporting document. To ensure confidentiality, all identifiable patient and surgical personnel information was removed or anonymized by the researchers. Data extraction and verification were conducted by multiple investigators to ensure accuracy and consistency. Furthermore, two researchers independently classified the associated characteristics, and the classification criteria were reviewed and agreed upon through discussion with a third researcher. These measures helped ensure consistency, improve data reliability, and minimize potential biases in the initial classification process.

### 2.2. Category of URFO‐Related Events

#### 2.2.1. Definition of URFO‐Related Events

URFO‐related events are generally defined as the intraoperative loss of surgical instruments or materials that meet one or more of the following criteria: (1) the item remains unrecovered despite multiple systematic search efforts by the surgical team; (2) retrieval is achieved only through intraoperative radiographic screening; or (3) the item is ultimately located but exhibits evidence of structural damage or fragmentation. These events are considered clinically significant due to their potential to disrupt surgical workflow, prolong operative time, and pose risks to patient safety.

#### 2.2.2. URFO‐Related Events Management Process

Prior to cranial closure or the completion of the primary surgical procedure, the scrub nurse performs a routine count of all surgical instruments and materials—including cotton pads, sponges, and sutures—and verifies their quantity and integrity in collaboration with the surgeon and circulating nurse. If any potential loss is detected, an immediate and systematic search of both sterile and nonsterile areas is initiated, with a minimum of three comprehensive attempts made to locate the missing item.

• For radiopaque items, intraoperative bedside radiography is performed in coordination with radiology staff to determine whether the item remains within the surgical field.
• For nonradiopaque items, the surgeon conducts a meticulous reinspection of the operative site to confirm the absence of retained materials, after which the event is documented and reported as necessary.

If item loss or breakage occurs during use—for example, a suture needle is dropped, a hemostatic clip is dislodged, or an instrument tip breaks during surgery—search protocols are activated immediately, and the case is managed according to standardized institutional procedures. Even when the missing or damaged item is subsequently located or retrieved, the incident is still required to be documented and reported through the hospital adverse event management system to facilitate ongoing safety surveillance and quality improvement.

#### 2.2.3. Cotton Material

A total of 132 cotton material loss events were reported (Figure 1H,I), including losses, damages, knot losses, and separations. These events involved three categories of cotton materials based on their size and structure. All cotton materials were equipped with radiopaque markers for intraoperative detection. The classification is based on the size and common clinical use of the cotton materials.

#### 2.2.4. Needle

A total of 156 needle‐related events were reported (Figure 1D,E). These were categorized based on needle type and intended surgical use. Although no URFO‐related events were recorded for 5‐0, 6‐0, or 7‐0 needles individually, they were grouped into the same category as vessel anastomosis needles due to their shared function and surgical application.

#### 2.2.5. Device Fragments

A total of 151 cases of device fragment loss events were recorded (Figure 1F,G). Device fragments are defined as the loss of broken parts of surgical instruments, including the breakage of sharp instrument tips and drill tips. These are categorized as instrument‐related issues and do not include the loss of cotton pads or surgical needles.

#### 2.2.6. Device Loss

**FIGURE 2:**
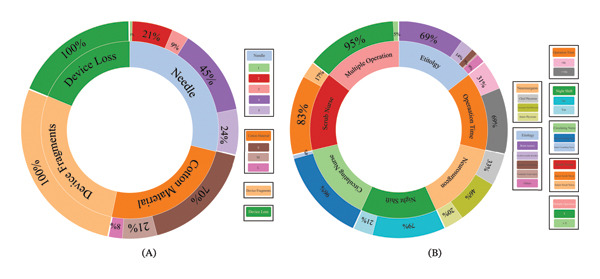
Classification and distribution of URFO‐related events and associated perioperative characteristics in neurosurgery. (A) Proportions of URFO‐related events subcategories. (B) Proportions of associated characteristics.

A total of 104 cases of device loss events were reported (Figure 1B,C). Device loss is defined as the complete loss of surgical items, including titanium screws, titanium plates, and scalp clips, but does not include the loss of cotton pads or surgical needles (Figure 2A).

### 2.3. Statistics

To further explore the factors associated with different categories of URFO‐related events, a multinomial logistic regression analysis was performed, with the four URFO‐related event types specified as outcome categories and one category selected as the reference group. The model was used to estimate the associations between perioperative variables and each URFO‐related event type based on regression coefficients and *p* values, thereby describing the distributional relationships between clinical factors and event categories.

All statistical analyses were performed within the subset of URFO‐related events; thus, the multinomial logistic regression was used to examine differences in event‐type distribution rather than to identify associated characteristics for URFO occurrence in the entire neurosurgical cohort.

Because this was a retrospective study including all eligible URFO‐related events during the study period, no a priori power analysis was performed. The sample size was considered adequate for multivariable logistic regression according to the events‐per‐variable principle, with 543 events available for seven candidate predictor variables, yielding an EPV of approximately 77.6. Prior to multivariable analysis, associations between categorical variables were assessed using chi‐square tests. Variables with statistically significant associations (*p* < 0.05) were considered for inclusion in the regression model. In addition, Cramer’s V was calculated to evaluate the strength of association for categorical variables. These procedures were applied to support variable selection and to describe the magnitude of associations, rather than to infer causality.

## 3. Results

**FIGURE 3:**
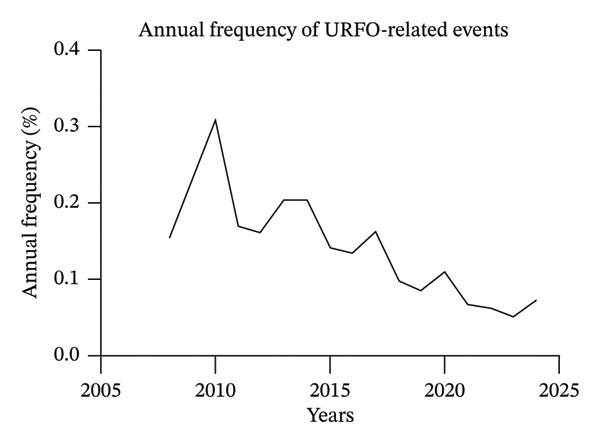
Annual Frequency of URFO‐related events in neurosurgical procedures from 2008 to 2024.

The overall frequency of URFO‐related events was 12.2 per 10,000 neurosurgical procedures (543/444,252) during the study period. The corresponding type‐specific rates per 10,000 procedures were as follows: cotton material (2.97), needle (3.51), device fragments (3.40), and device loss (2.27). Temporal trends over the past 16 years are shown (Figure 3; Table S1). A list of common items used in neurosurgical operating rooms is provided in the supporting document (Figure S1).

### 3.1. Associated Characteristics of URFO‐Related Events Based on Sankey Diagram Analysis

**FIGURE 4:**
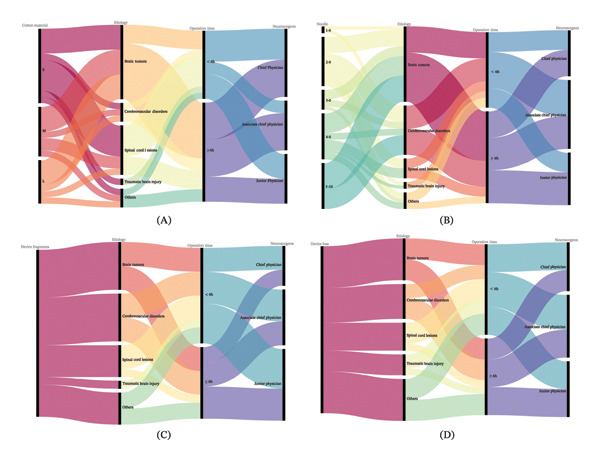
Sankey diagram of neurosurgical URFO‐related events and perioperative variables. (A) Cotton material. (B) Needle. (C) Device fragments. (D) Device loss.

The Sankey diagram illustrates the perioperative characteristics associated with different URFO‐related event types. Most URFO‐related events were observed in neurosurgical tumor procedures. For these Level IV surgeries, the primary surgeons were mainly chief or associate chief physicians. Circulating nurses were generally senior nurses due to structured training pathways under scrub nurse supervision, while scrub nurses were more often junior staff (Figure 2B). Cotton material events were distributed across multiple etiologies. These events were more frequently observed in long‐duration procedures and were associated with varying levels of surgical seniority. In particular, procedures lasting ≥ 6 h were more commonly performed by junior neurosurgeons (Figure 4A).

Needle‐related events showed variation across different types of needles, including muscle suture needles, subcutaneous suture needles, and vascular anastomosis needles. A relatively higher proportion of these events occurred in brain tumor and cerebrovascular disease surgeries. Longer operative duration appeared to coincide with a greater proportion of such events, whereas cases involving senior neurosurgeons were less frequently represented (Figure 4B).

Device fragment events were primarily concentrated in patients with brain tumors and spinal cord injuries, with a higher proportion of long‐duration surgeries and less experienced surgeons, suggesting that procedural complexity and experience level may be relevant contextual factors (Figure 4C).

Device loss events were mainly observed in brain tumor and cerebrovascular disease cases and were more common in long‐duration surgeries. Cases involving chief and associate chief physicians were relatively more common, potentially reflecting the higher complexity of these surgical procedures (Figure 4D).

In summary, long‐duration neurosurgical tumor procedures constitute a major setting in which URFO‐related events are concentrated. Cotton materials and 4‐0 sutures appeared more frequently involved in complex procedures. These findings highlight the importance of strengthening standardized counting procedures and perioperative item management. The Sankey diagram provides a visual overview of the distributional relationships between URFO‐related events and perioperative variables, offering insights for improving operating room workflow standardization and item safety management.

### 3.2. Multinomial Logistic Regression Analysis of Factors Associated With URFO‐Related Events

We performed a multinomial logistic regression analysis to examine factors associated with different URFO‐related event types. Given the relatively balanced distribution across the four URFO categories, each category was alternatively set as the reference group to explore the relative associations between cotton material, needle, device fragments, and device loss and perioperative factors.

The independent variables included etiology, operation time, neurosurgeon, night shift, circulating nurse, scrub nurse, and multiple operations. To ensure model stability and reliable parameter estimation, clinically representative categories with sufficient sample sizes were selected as reference groups: etiology (brain tumor), operation time (< 6 h), neurosurgeon (chief physician), night shift (non‐night shift), circulating nurse (senior), scrub nurse (junior), and multiple operations (no).

Due to the small sample size of traumatic brain injury cases (Etiology 4), this category was excluded from the regression analysis to avoid model instability and inflated standard errors. Detailed regression coefficients and *p* values are provided in the supporting document (Table S2–5).

**FIGURE 5:**
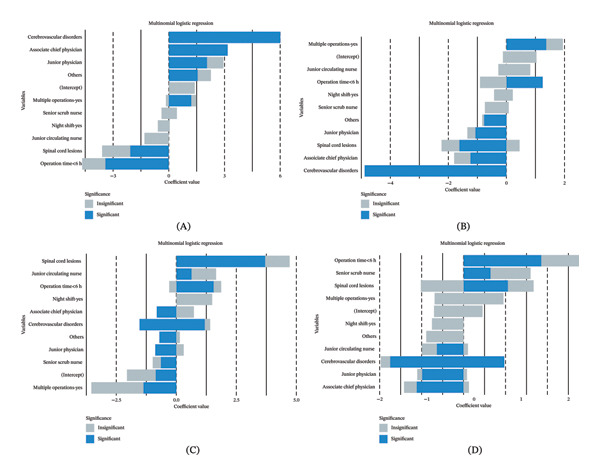
Multinomial regression analysis of factors associated with different types of URFO‐related events. Blue indicates statistical significance; gray indicates nonsignificant results. (A) Cotton material as the reference category. (B) Needle as the reference category. (C) Device fragments as the reference category. (D) Device loss as the reference category.

Overall, the results suggest that perioperative factors such as etiology, operation duration, and staff characteristics were differentially associated with the distribution of URFO‐related event types (Figure 5A–D). The full regression results can be found in the supporting document.

### 3.3. Correlation Analysis of Factors Associated With Different Types of URFO‐Related Events

**FIGURE 6:**
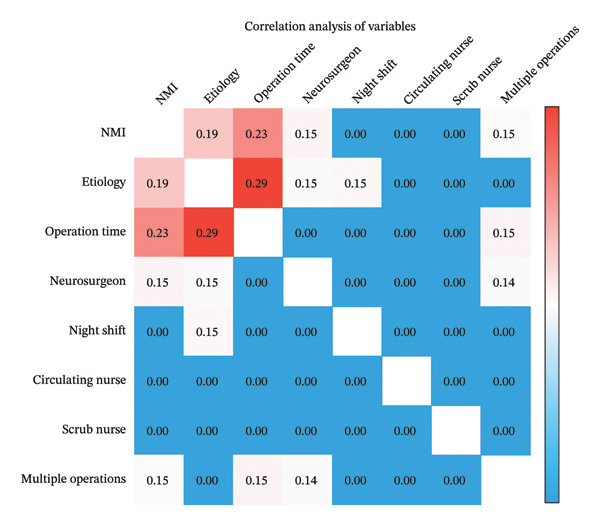
Correlation analysis of perioperative variables associated with different URFO‐related events.

The correlation analysis showed that URFO‐related events were mainly associated with etiology and operation time, with correlation coefficients of 0.19 and 0.23, respectively, suggesting that variations in surgical etiology and procedure duration may be related to different URFO‐related events. A weak correlation was observed between URFO‐related events and the neurosurgeon category (*V* = 0.15), In contrast, nursing‐related variables including circulating nurses and scrub nurses, demonstrated minimal associations with URFO‐related event categories (*V* < 0.1). In conclusion, etiology and operation time were the variables most strongly associated with the distribution of different URFO‐related events (Figure 6).

## 4. Discussion

Due to an aging population, increasing comorbidities, and advances in surgical techniques, the clinical management of hospitalized patients has become more complex [8–10]. Perioperative management of surgical patients is crucial. Existing studies on perioperative adverse events mainly focus on drug reactions, hospital infections, postoperative complications, and hospital‐acquired conditions (pressure ulcers, lower extremity venous thromboembolism, falls, etc.) [11, 12]. Currently, most research on “Near‐Miss” events are concentrated in the fields of obstetrics and neonatology [13, 14], likely due to the high monitoring needs during the perinatal period and the potential impact on maternal and neonatal safety. There is limited research focusing on intraoperative URFO–related events. In neurosurgery, the loss of intraoperative items can not only result in foreign body retention, leading to inflammatory foreign body reactions [15], but also increase the risk of infection, inflammation, and the need for secondary surgeries. Furthermore, the time spent searching for lost items during surgery can prolong anesthesia, increasing the likelihood of neurological damage and postoperative infections [16–18].

Patient safety, operating room management, and perioperative nursing have consistently remained central themes in healthcare research [19]. Over time, the focus of research has evolved from individual‐level factors, such as staff competence, teamwork, and attitudes, toward broader system‐ and organizational‐level issues emphasizing safety management, organizational culture, and quality improvement [20, 21]. Our findings support the importance of competency‐based staffing and standardized counting procedures, particularly in complex and prolonged neurosurgical operations. Improving nursing management capability can be achieved through multiple approaches, among which close collaboration between operating room nurses and other healthcare practitioners is particularly important [22]. Ensuring operating room safety relies not only on individual professional competence but also on effective interdisciplinary communication, adherence to standardized guidelines, and adequate organizational support, all of which are equally applicable to the management of URFO‐related events [23]. The implementation of structured risk management strategies in the operating room may contribute to measurable improvements in nursing quality, strengthen staff risk awareness and safety practices, and enhance patient satisfaction [24, 25]. Collectively, these findings support the broader integration of risk‐based perioperative nursing strategies into operating room management systems to promote safer, higher‐quality surgical care and strengthen the overall culture of patient safety.

Our study analyzed a total of 543 URFO‐related events over 16 years, representing, to the best of our knowledge, the longest duration and largest case series of neurosurgical URFO‐related events [2, 3, 26, 27]. The frequency of URFO‐related events in neurosurgery was found to be 12.2 per 10,000 procedures (0.122%). The higher observed frequency may be largely attributed to our broader operational definition of URFO‐related events, which includes not only irretrievable items but also those located through intraoperative radiographic imaging or retrieved in a damaged or fragmented condition. Overall, the frequency has shown a downward trend over time (Figure 3), which may reflect advancements in surgical techniques and improvements in nursing management systems. In addition, the widespread use of radiopaque materials has enabled earlier detection of such events, providing an additional safeguard for surgical safety [28]. Our findings further showed that URFO‐related events were more frequently observed in brain tumor surgeries, procedures lasting ≥ 6 h, surgeries involving associate chief or junior physicians, and cases involving lower‐seniority nursing staff. These findings provide important insights into the perioperative characteristics associated with URFO‐related events, which can inform targeted preventive measures to reduce their frequency.

Overall, URFO‐related events were more frequently observed in complex surgical contexts, including brain tumor procedures, prolonged operations, and cases involving less experienced surgical and nursing staff. These findings suggest that procedural complexity, workload intensity, and team experience may jointly influence intraoperative item management. Small and frequently used materials, as well as delicate surgical instruments, may be particularly vulnerable to misplacement or loss under conditions of high operative demand and divided attention.

From a systems perspective, these results support a shift from individual‐focused explanations toward structured safety management strategies. Standardized counting procedures, competency‐based staffing allocation, and structured intraoperative communication mechanisms may help reduce variability in practice. In addition, targeted training for junior surgeons and nurses, particularly in instrument handling and tracking, may enhance procedural consistency. The integration of double‐check systems and emerging tracking technologies may further improve reliability in complex surgical environments.

Importantly, our findings align with the broader transition in perioperative nursing research from individual‐level factors to system‐level safety governance. Strengthening organizational safety structures, promoting interdisciplinary collaboration, and implementing standardized protocols may therefore represent key strategies for reducing URFO‐related events and improving overall surgical safety culture.

In addition, due to the nature of the adverse event reporting system, our dataset did not allow for precise differentiation among specific subtypes of URFO‐related events, such as recovered before closure (near‐miss), “lost then found” outside the patient, and breakage or fragmentation without retention. Importantly, all URFO‐related events included in this study were ultimately confirmed to have no retained foreign objects within the patient. This limitation may affect the granularity of event classification and interpretation of findings.

Future multicenter studies with more comprehensive datasets are warranted to further refine event classification, validate these findings, and support the development of standardized perioperative safety management strategies.

## 5. Conclusions

This study analyzed 543 URFO‐related events over a 16‐year period, representing, to the best of our knowledge, the largest and longest case series focused on neurosurgical URFO‐related events. To further characterize the patterns of URFO‐related events, we conducted a multinomial logistic regression analysis of clinical, procedural, and perioperative team characteristics. Our findings showed that URFO events were more frequently observed in brain tumor surgeries, procedures performed by associate chief or junior surgeons, operations lasting ≥ 6 h, and cases involving less experienced nursing staff (e.g., circulating nurses). In addition, we established a clear and comprehensive definition of URFO‐related events and implemented a standardized intraoperative management protocol. The subsequent decline in URFO‐related events at our institution over time suggests the potential effectiveness of this standardized perioperative management approach, supporting its value in improving surgical safety and nursing quality.

## 6. Implications for Nursing Management

The findings of this study support several targeted implications for perioperative nursing management. First, the higher frequency of URFO‐related events in brain tumor surgeries and in procedures lasting ≥ 6 h highlights the need for enhanced nursing vigilance and strengthened counting protocols in complex and prolonged neurosurgical procedures. In such cases, dedicated reinforcement of instrument and material tracking throughout the operation may be particularly important.

Second, the increased occurrence of URFO‐related events in surgeries performed by associate chief or junior surgeons suggests that staffing allocation should be adjusted according to surgical experience level. Specifically, assigning more experienced circulating and scrub nurses to cases involving less experienced surgeons may help reduce the risk of retained or mismanaged items.

Third, the association between URFO‐related events and less experienced nursing staff further emphasizes the importance of competency‐based staffing and structured intraoperative supervision. Targeted training programs focusing on counting accuracy and instrument handling should be prioritized for junior nursing staff.

The observed temporal decline in URFO‐related events following the implementation of standardized perioperative management measures may suggest a potential benefit of system‐level safety interventions.

Overall, these findings suggest that risk‐stratified nursing management strategies tailored to surgical complexity, duration, surgeon experience, and nursing competency may effectively enhance perioperative safety.

## Author Contributions

Chao Du contributed to the study design, data analysis, and wrote the manuscript. Shuo Zhang was involved in study design and data collection. Xiaonan Liu contributed to the study design. Dan Liu, Jing Li, Wei Wang, and Hongling Jiao contributed to data collection and processing. Xiaonan Liu secured project funding. Chao Du, Shuo Zhang, and Xiaonan Liu reviewed and edited the intellectual content.

## Funding

This study was supported by the Young Investigator Nursing Research Fund of Beijing Tiantan Hospital (Grant No. 2020‐YQN‐09).

## Disclosure

All authors gave final approval for this version to be published. All authors read and approved the final manuscript.

## Ethics Statement

This study was approved by the Ethics Committee of Beijing Tiantan Hospital, Capital Medical University (Approval No. KY2023‐260‐02). All patients signed an informed consent form upon hospital admission, which included consent for the use of their anonymized clinical data for research purposes.

## Conflicts of Interest

The authors declare no conflicts of interest.

## Supporting Information

Additional supporting information can be found online in the Supporting Information section.

## Supporting information


**Supporting Information** The supporting document contains supporting definitions of URFO‐related event classifications, including retained material types, surgical etiologies, surgeon and nursing staff classifications, operation characteristics, and multiple‐operation criteria. It also provides detailed multinomial logistic regression analyses evaluating factors associated with different categories of URFO‐related events. (1) Table S1: Annual operation volume and frequency of URFO‐related events in neurosurgery. (2) Table S2: Multivariate logistic regression analysis of coefficients with cotton material as the reference category. (3) Table S3: Multinomial logistic regression analysis with needle‐related events as the reference category. (4) Table S4: Multinomial logistic regression analysis with device fragment–related events as the reference category. (5) Table S5: Multinomial logistic regression analysis with device loss–related events as the reference category. (6) Figure S1: Representative items commonly used in neurosurgical operating rooms: (A) titanium screws, (B) cotton stick, (C) cotton pads, (D) suction device, (E) scalp clips, (F) bone drill, and (G) bone cutter burrs. (7) STROBE checklist cohort.

## Data Availability

The datasets generated during the current study are available from the corresponding author upon reasonable request.
