# Supplementary material for: Perioperative Nursing Management of Unintended Retained Foreign Object–Related Events in Neurosurgery: Insights From a 16‐Year Study in China
Source: J Nurs Manag. 2026 Aug 12;2026:5532006. doi: 10.1155/jonm/5532006 (PMC13463234; doi:10.1155/jonm/5532006)
Supplement: Supplementary file 1 [file JONM-2026-5532006-s001.zip › Figure S1.pdf]

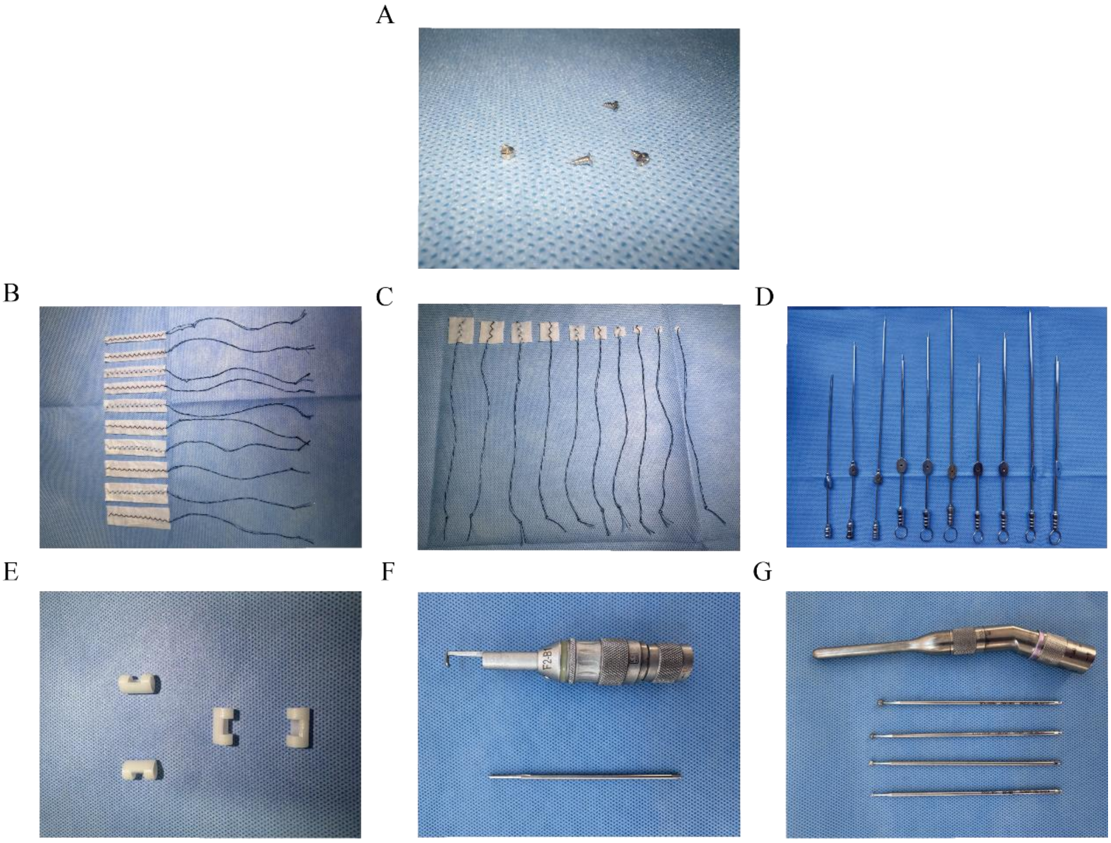

Fig. S1 Common Items in Neurosurgical Operating Rooms. (A) Titanium Screws. (B) Cotton Stick. (C) Cotton Pads. (D) Suction Device. (E) Scalp Clips. (F) Bone Drill. (G) Bone Cutter Burrs.
