# Supplementary material for: Perioperative Nursing Management of Unintended Retained Foreign Object–Related Events in Neurosurgery: Insights From a 16‐Year Study in China
Source: J Nurs Manag. 2026 Aug 12;2026:5532006. doi: 10.1155/jonm/5532006 (PMC13463234; doi:10.1155/jonm/5532006)
Supplement: Supplementary file 1 [file JONM-2026-5532006-s001.zip › Supplementary Document.docx]

**Classification definition of URFO-related events**

1. **Cotton Material**

- **S-type**: non-foldable cotton pads (30 mm × 25 mm, *n* = 93, 70%)
- **M-type**: non-foldable cotton sliver (80 mm × 25 mm, *n* = 28, 21%)
- **L-type**: foldable gauze materials (*n* = 11, 8%), consisting of:
  - Gauze strips (folded size: 30 mm × 100 mm, four layers)
  - Gauze pads (folded size: 80 mm × 80 mm, four layers)

**2. Needle**

- 1-0 (muscle suture needles): n = 2 (1%)
- 2-0 (subcutaneous tissue suture needles): n = 33 (21%)
- 3-0 (skin suture needles): n = 14 (9%)
- 4-0 (dura mater suture needles): n = 70 (45%)
- 5-0 to 10-0 (vessel anastomosis needles): n = 37 (24%)

**Classification of Etiology**

The causes of neurosurgical conditions in this study were classified into five categories:

- **Brain Tumors**: Refers to space-occupying lesions that are either primary or metastatic to the brain, requiring craniotomy or endoscopic surgical intervention.
- **Cerebrovascular Disorders**: Includes cerebrovascular conditions necessitating surgical treatment, such as hemorrhagic stroke, cerebral aneurysms, and arteriovenous malformations.
- **Spinal Cord Lesions**: Encompasses pathologies involving structural damage or abnormalities within or surrounding the spinal cord, including traumatic spinal cord injury, intramedullary lesions, and syringomyelia.
- **Traumatic Brain Injury**: Refers to impaired brain function resulting from external mechanical forces—such as collisions, falls, or motor vehicle accidents—causing damage to the head.
- **Others**: Includes less common surgical procedures, such as radical resection of epileptogenic foci, cranioplasty, wound debridement, decompressive craniectomy for malignant cerebral infarction, and specialized nerve reconstruction procedures (e.g., hypoglossal-facial nerve anastomosis with pre-modified sural nerve grafts, facial nerve decompression, ventriculoperitoneal shunt for congenital encephalocele and obstructive hydrocephalus).

**Classification of Neurosurgeons**

Neurosurgeons were categorized into three professional levels according to their academic background and clinical experience:

- **Chief Physician**: Holds a bachelor's degree or higher and has served as an Associate Chief Physician for at least five years.
- **Associate Chief Physician**: Holds a bachelor’s degree or above and has attained mid-level seniority as an Associate Chief Physician.
- **Junior Physician**: Includes residents and attending physicians who possess fundamental medical training and clinical skills. They are primarily responsible for the diagnosis and treatment of common cases, assist in surgeries, and contribute to inpatient management. Their professional development progresses through continuous education and clinical practice.

**Operation Time**

Operation time was categorized into two groups:

- ≥ 6 hours
- < 6 hours

**Night Shift Definition**

A night shift was defined as any surgical procedure during which the instrument nurse assumed responsibility after 18:00.

**Classification of Circulating and Scrub Nurses**

Circulating and scrub nurses were both stratified into two levels:

- **Senior**: Defined as nurses with five or more years of neurosurgical operating room experience.
- **Junior**: Defined as nurses with less than five years of relevant experience.

**Definition of Multiple Operations**

Multiple operations were defined as secondary or repeated neurosurgical procedures. These include surgeries for tumor recurrence, postoperative intracranial hemorrhage, decompressive craniectomy, or other indications requiring subsequent surgical intervention on the brain.

**The Results of Multinomial Logistic Regression Curve Analysis**

**1.Cotton material as the reference category**

Compared with cotton material–related events, brain tumor surgeries were more frequently associated with needle-related (Coeff = 2.723, p < 0.05), device fragment–related (Coeff = 1.532, p < 0.001), and device loss–related events (Coeff = 1.751, p < 0.001). Procedures performed by associate chief physicians and junior physicians were also significantly associated with needle-related and device loss–related events. In contrast, operation time ≥6 hours showed significant associations mainly with device fragment–related and device loss–related events.

**2.Needle-related events as the reference category**

When needle-related events were used as the reference category, brain tumor surgeries remained significantly associated with cotton material–related (Coeff = −2.724, p < 0.05) and device fragment–related events (Coeff = −1.190, p < 0.001). Operation time ≥6 hours was significantly associated with needle-related events relative to cotton material–related events (Coeff = 1.252, p < 0.05), whereas no significant associations were observed for device fragment–related or device loss–related events. Procedures performed by associate chief physicians and junior physicians also showed significant associations across comparisons involving needle-related events.

**3.Device fragment–related events as the reference category**

Compared with device fragment–related events, brain tumor surgeries were more frequently associated with cotton material–related (Coeff = 1.532, p < 0.001), needle-related (Coeff = 2.723, p < 0.05), and device loss–related events (Coeff = 1.751, p < 0.001). Associate chief physicians were significantly associated with comparisons involving device loss–related events (Coeff = 0.813, p = 0.005), whereas no significant association was observed for junior physicians. In addition, lower-seniority circulating nurses were significantly associated with device loss–related events (Coeff = 0.636, p = 0.025), suggesting that perioperative team experience may influence intraoperative item management.

**4.Device loss–related events as the reference category**

When device loss–related events were used as the reference category, brain tumor surgeries remained significantly associated with cotton material–related and needle-related events across multiple comparisons. Procedures lasting ≥6 hours showed significant associations in comparisons involving device loss–related events (Coeff = −1.853, p < 0.001). In addition, surgeries performed by associate chief physicians and junior physicians were significantly associated with device loss–related events (Coeff = 1.116, p = 0.002; Coeff = 0.990, p = 0.003). Lower-seniority circulating nurses also demonstrated significant associations with device loss–related events (Coeff = 0.636, p = 0.025), further supporting the influence of perioperative staffing experience on URFO-related event distribution.
